# Internal jugular vein tumor thrombus in papillary thyroid cancer: our institution’s experience and a systematic review of the literature

**DOI:** 10.3389/fendo.2025.1514455

**Published:** 2025-02-25

**Authors:** Bryce J. Laurin, Robert Ballard, Ifthikar Malik, Janeil Mitchell

**Affiliations:** ^1^ School of Medicine, Medical College of Wisconsin, Milwaukee, WI, United States; ^2^ Department of Vascular Surgery, Fox Valley Surgical Specialists, Appleton, WI, United States; ^3^ Division of Endocrinology, Fox Valley Surgical Specialists, Appleton, WI, United States; ^4^ Division of Endocrine Surgery, Department of General Surgery, Fox Valley Surgical Specialists, Appleton, WI, United States

**Keywords:** thyroid cancer, *BRAFV600E*, metastatic papillary thyroid cancer, tumor thrombus, internal jugular vein thrombus

## Abstract

Papillary thyroid tumor thrombosis of the internal jugular vein (IJV) is a rarely observed phenomenon with fewer than 30 cases reported to date. The clinical features and underlying pathogenesis of tumor thrombosis are not well-elucidated. A PRISMA-compliant systematic review was conducted, yielding 20 studies eligible for analysis. Additionally, we describe a case of papillary thyroid cancer (PTC) tumor thrombus involving the IJV with solitary metastasis to the ipsilateral kidney. The majority of patients in the cohort presented in an asymptomatic state (n = 14) with variable timepoints in diagnosis: preoperative (n = 9), intraoperatively (n = 1), and postoperative period (n =11), up to 30 years post-thyroidectomy. Primary tumor sizes ranged widely, with a mean of 4.22 cm ± 2.64cm. Most patients (85.7%) presented with nodal involvement and a few (n =4) had distant metastases with pulmonary involvement most commonly reported. Open tumor thrombectomy was performed in 10 (52.6%) cases and extensive vascular reconstruction was required in 8 (42%). Adjuvant treatment including radioactive iodine ablation (36.8%) and external beam radiation (21.1%) was also employed. Patient clinical factors, presentation, diagnosis, and management of PTC vascular tumor thrombus are heterogeneous. Tumor thrombus occurred in patients with solitary, small primary tumors and patients with heavy locoregional disease burden and presents as isolated and extensive thrombotic burden, the latter requiring complex open cardiovascular reconstruction in some patients. The rarity of the disease and diverse clinical presentation reporting remains a challenge in the understanding of pathogenesis, optimal management, and outcomes in PTC-related thrombosis.

## Introduction

In 2025, over 40,000 individuals are estimated to be diagnosed with papillary thyroid cancer (PTC) in the United States, accounting for 2.2% of all new cancer cases in the country (1). The majority of PTC disease remains localized within the thyroid with a very favorable prognosis, over 98% overall survival at 5 years (1, 2). Microscopic extrathyroidal growth can be observed in as many as a third of cases, however, grossly locally-invasive disease with involvement of adjacent tissue (i.e., strap muscle, trachea, esophagus, and carotid artery) is uncommon, occurring in just under 5% of cases (3, 4). Involvement of adjacent tissue is associated with poorer outcomes including mortality (3, 5, 6). The gross direct extension of the tumor into large vessels or tumor thrombosis by PTC is rare, <1% of cases (7). Fewer than 30 cases of PTC with tumor thrombosis of regional vasculature [internal jugular vein (IJV), superior vena cava (SVC), and ascending aorta] have been reported to date (8–26). To our knowledge, there have only been four other reported cases of tumor thrombosis and distant metastasis occurring in the same patient (13, 23, 24, 27).

A mechanism for the pathogenesis of PTC tumor thrombus has not been determined. A tumor thrombus, presence of intraluminal carcinoma, is distinct from direct vascular wall invasion and the pathogenesis likely differs from that seen in other carcinomas (i.e., renal cell carcinoma) observed to develop malignant thrombosis. Researchers have postulated that patients with PTC vascular thrombosis may be susceptible due to a secondary hypercoagulable state from underlying malignancy and, if present, hypothyroidism (28–31). Circulating PTC cells passing through the venous system via venous connections with previously invaded lymph nodes are thought to initiate the development of tumor thrombus (19).

Risk factors for advanced and metastatic PTC include male sex, advanced age, high-risk (i.e. *TERT* promoter mutations) or multiple oncogenic mutations, aggressive variants, extrathyroidal extension, and lymph node metastasis at initial examination (32–34) and were examined in our series. Independent risk factors for the development of tumor thrombi to inform potential pathogenesis and direct further mechanistic research, is unknown.

As a unique contribution to the existing small body of literature, we present a case of pathologically confirmed PTC with a tumor thrombus present in the IJV and metastasis to the ipsilateral kidney. We analyzed this case in the context of a systematic literature review of tumor thrombi in PTC with an examination of clinical features or potential risk factors and treatment reported.

## Methods

A PubMed search was conducted following the guidelines of the Preferred Reporting Items for Systematic Reviews and Meta-Analyses (PRISMA) for any resulting articles under the search criterion “papillary thyroid cancer AND tumor thrombus.” Separate searches for “papillary thyroid cancer AND direct vascular extension,” and “papillary thyroid cancer AND direct vascular extension AND venous invasion” did not reveal any new studies describing tumor thrombosis. Inclusion criteria included adult or pediatric patients with a pathologically confirmed PTC tumor thrombus within any vasculature structure, either pre-, intra-, or post-operatively, with PTC or PTC variant subtypes, including follicular-variant PTC primary tumors. Exclusion criteria included articles with cases reporting microscopic angioinvasion rather than IJV thrombosis, pathologies other than PTC (including PTC with anaplastic thyroid carcinoma), articles unavailable in full text, and non-English language articles. Primary studies such as case reports were included. Secondary studies such as systematic reviews and meta-analyses were excluded unless new patients were presented. One author (BJL) performed the initial screening of all titles and abstracts. Studies that either seemed appropriate for inclusion or those that could not clearly be excluded on title and abstract alone were carried forward for further full-text evaluation.

**Figure 1:**
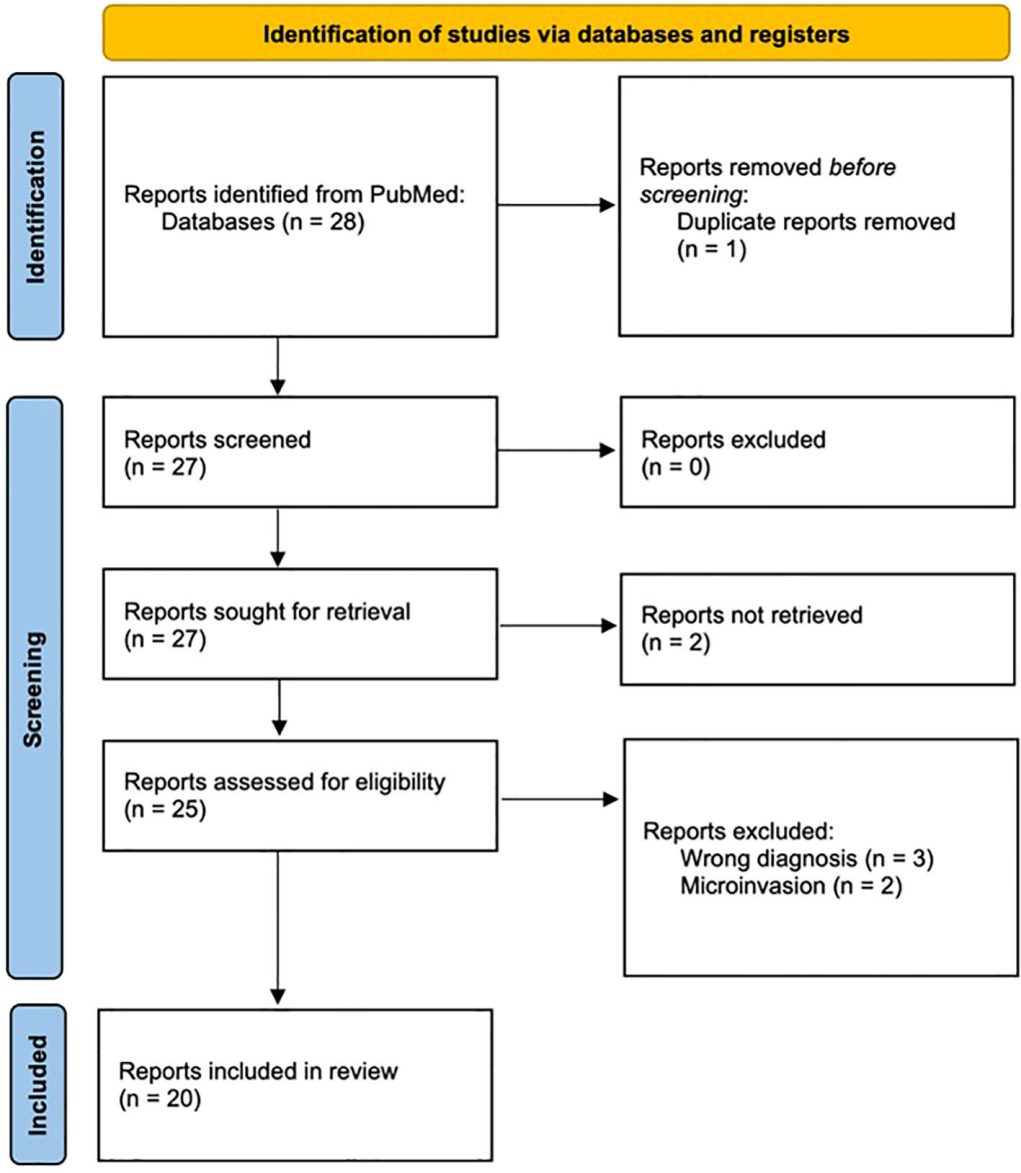
PRISMA flow diagram of literature screening and exclusion criteria. BMJ 2021;372:n71.

The PubMed search yielded a total of 28 articles. A duplicate article was excluded. Full-text reports were not retrievable for two studies. Two articles were excluded due to reported PTC microscopic angioinvasion. Three articles were additionally excluded as thrombosis occurred in pathologies other than PTC. The search query was repeated and did not produce any new articles. A total of 20 studies were deemed eligible and included in this systematic review along with our institution’s case for total of 21 unique patients (**Figure 1**).

Patient demographics, clinical attributes, tumor characteristics, diagnostic evaluations, treatment interventions, length of longitudinal follow-up, and reported mortality were extracted from each systematic review article and our case and summarized in **Table 1**.

**Table 1:** Summary of each case included in the analysis.

Source	Age	Sex	Surgical histopathology	Thyroid function	Preoperative symptoms	Primary tumor size (cm)	Tumor thrombus diagnosis	Tumor thrombus diagnostic modality	Vascular thromus site	Pre-thrombectomy serum Tg (ng/ml)	Extrathyroidal growth	Central nodal metastasis	Lateral nodal metastasis	Distant metastasis	Treatment	Post-thrombectomy serum Tg (ng/ml)	Post-thrombectomy follow-up and outcome
Agrawal (2009) (8)	48	M	FV-PTC	−	None	−	Postop	I-131 NM	IJV, SCV	>800	−	−	−	N	IJV thrombectomy with partial SCV resection & SVC repair	121	6-month DFS
Al-Jarrah (2014) (9)	62	F	PTC	Euthyroid	Thoracic outlet	5	Preop	US	IJV	>138	Anterior tracheal wall	Y	N	N	IJV thrombectomy, RAI, RT	−	Alive at 16-month follow-up
Babu (2012) (10)	68	F	PTC	−	None	−	Postop	CT	IJV	−	N	Y	N	N	IJV thrombectomy w/ distal and proximal IJV, BCV, and SCV resection	−	Survived procedure
Dikici AS (2015) (11)	52	F	PTC	Euthyroid	None	5.5	Postop	US/CT	IJV	−	N	Y	−	N	IJV thrombectomy w/ IJV resection and reconstruction w/ saphenous vein	−	Survived procedure
Gui Y (2022) (12)	45	F	PTC	Euthyroid	None	0.9	Preop	US	Middle thyroid vein	Normal	N	N	N	N	Middle thyroid vein resection	−	6-month DFS
Hasegawa, S (2002) (13)	78	F	PTC	−	Severe and uncontrollable neck pain	−	Preop	CT/MRI/US/PET	IJV, SVC, atrium	−	N	N	N	Lung	IJV, SVC, atrium resection	−	Died on POD 36, respiratory failure
Ingle SA (2004) (14)	−	−	PTC	−	SVC syndrome	−	Preop	−	azygous, IJV	−	−	−	−	−	−	−	−
Jafari F (2024) (15)	40	F	FV-PTC	Hypothyroidism	None	3	Postop	US	IJV	Normal	N	Y	Y	N	IJV thrombectomy, RAI, RT	>100	Significant post-surgical residual disease and logoregional node involvement
Jain, P. V (2019) (16)	44	F	PTC	−	−	−	Postop	I-131 NM	IJV, SVC	−	N	Y	N	N	patient defaulted on surgery	−	−
Koike, E (2002) (17)	26	F	PTC (thrombus)	−	None	7.8	Postop	I-131 NM	BCV, SVC	19,800	Tracheal wall	Y	N	N	BCV thrombectomy, RAI	−	8-month DFS
Motohashi, S (2005) (18)	64	F	PTC	−	SVC syndrome	−	Preop	CT/MRI	IJV, BCV, SVC, caval-atrial junction	38,000	N	Y	N	N	IJV, BCV, SVC resection w/reconstruction w/ PTFE graft, RAI	Normal	3-year DFS
Osborne RF (2011) (19)	79	F	FV-PTC	−	None	−	Preop	CT	IJV	Elevated	Adjacent soft tissue	−	−	N	IJV ligation	−	Survived procedure
Patel, P. C. (1997) (20)	79	F	PTC	Euthyroid	Venous enorgement	−	Intraop	−	Azygous, IJV, BCV to caval-atrial junction, pulmonary vein	−	Recurrent laryngeal nerve, anterior tracheal wall	−	−	N	Cavotomy and thrombectomy	−	Died
Rampelly S (2022) (21)	50	M	PTC	Euthyroid	None	8	Preop	US/CT	IJV	Normal	Sternocleiodmasteoid muscle	Y	N	N	IJV, SCM resection, RAI	−	Survived procedure, discharged
Sanioglu S (2009) (22)	64	M	PTC	−	Thoracic outlet	2	Preop	CT/TEE	Ascending aorta aneurysm	−	N	−	−	N	Ascending aortic thrombectomy	−	Alive at POD 12
Sezer H (2021) (23)	63	M	PTC	−	None	1	Postop	US	IJV	−	Y	Y	−	Brain, lung, bone	IJV resection, RAI, RT, CT	64.5	Died due to disease progression, time unspecified
Sirota DK (1989) (27)	61	F	PTC	−	None	−	Postop	−	axillary vein	−	−	−	−	Y	−	−	Died due to disease progression, time unspecified
Wada N (2009) (24)	74	F	PTC	−	None	−	Postop	CT	BCV, SVC	707	−	Y	−	Lung	BCV, SVC resection and bypass Construction with autologous graft	−	Died of disease 19 months postop
Wang Y (2022) (25)	24	M	PTC	−	None	−	Postop	PET-CT	BCV, SVC	−	N	−	−	N	BCV resection with SVC reconstruction	288	Died 1.5 years postop from respiratory failure
Yamagami Y (2008) (26)	74	M	PTC	−	None	2	Postop	CT/TEE	IJV, BCV, SVC, atrium	−	N	Y	N	N	Thrombectomy	−	7-month DFS
Current case	60	F	FV-PTC	Subclinical hypothyroidism	None	7	postop	US	IJV, BCV	4,527	Recurrent laryngeal nerve	Y	Y	Kidney	IJV thrombectomy and resection, RAI, RT, partial nephrectomy	450	2-year DFS

Studies are organized in alphabetical order by first author’s last name. M, male; F, female; PTC, papillary thyroid cancer; FV, follicular variant; mo, month; yr, year; Tg, thyroglobulin; SCV, subclavian vein; SVC, superior vena cava; cm, centimeter; CT, computed tomography; US, ultrasound; MRI, magnetic resonance imaging; FDG PET, fludeoxyglucose-18 positron emission tomography; TEE, transesophageal echocardiogram; WNL, within normal limits; LN, lymph node; BCV, brachiocephalic vein; RAI, radioactive iodine; RT, radiation therapy; CT, chemotherapy; POD, postoperative day; DFS, disease-free survival; NM, nuclear medicine; PTFE, polytetrafluoroethylene; SCM, sternocleidomastoid muscle.

## Case report results

**Figure 2:**
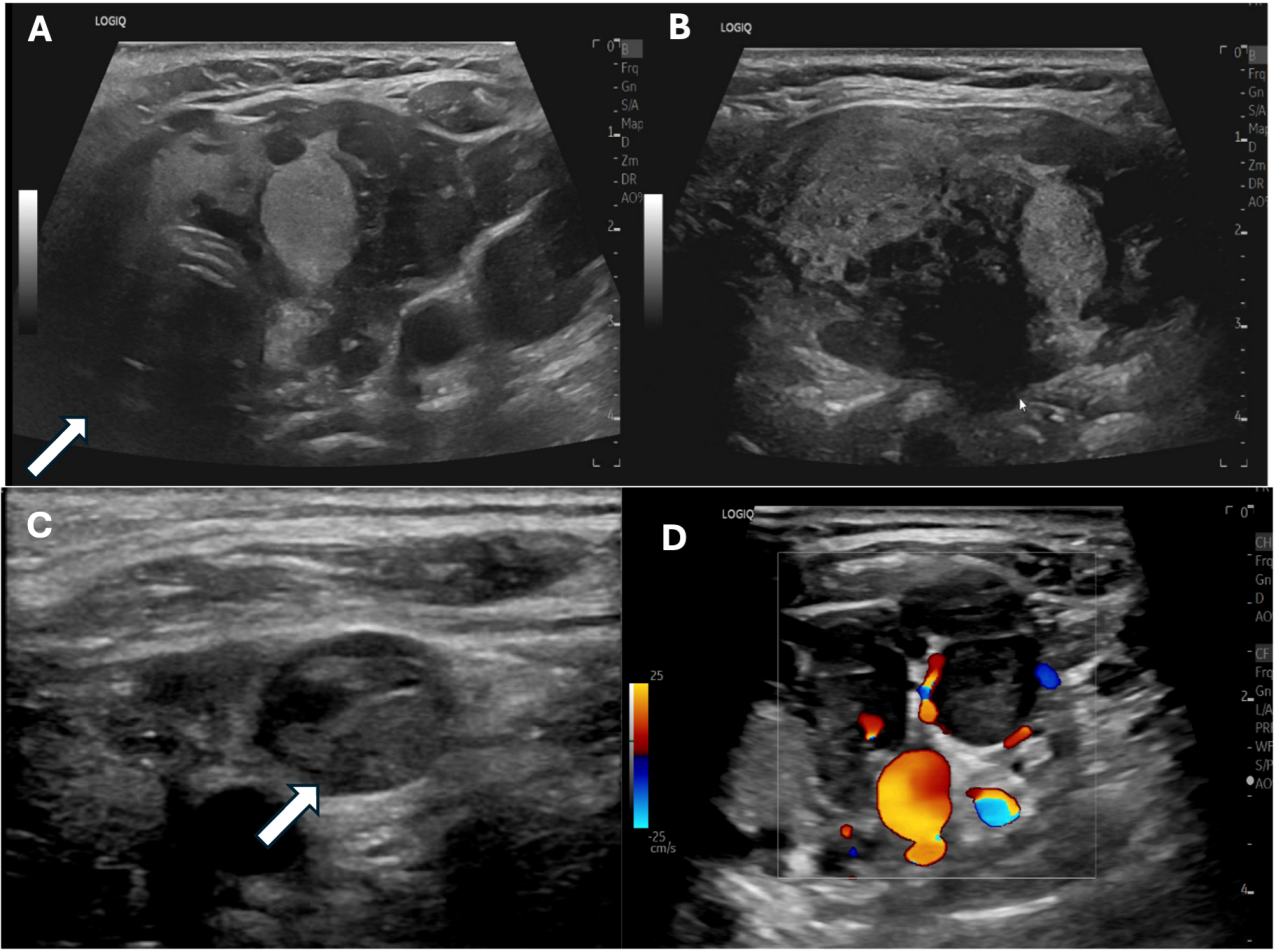
Preoperative sonogram of the left central neck **(A)**, transverse plane; **(B)**, longitudinal plane) demonstrating the multinodular left thyroid lobe with an arrow indicating the heterogeneous dominant left thyroid nodule replacing most of the left lobe, which returned benign cytology on fine needle aspiration. Preoperative neck ultrasound image **(C)** demonstrates the left internal jugular vein (white arrow) with intraluminal thrombus devoid of internal flow on doppler imaging **(D)**.

A 60-year-old woman with a remote 20-year history of tobacco use was found to have new thyroid gland enlargement during an annual exam with her primary care provider. She was not experiencing compressive symptoms, including globus, dysphagia, or hoarseness, nor neck or extremity swelling. She was subsequently found to have an elevated thyroid-stimulating hormone (TSH) level of 6.18 μIU/mL but normal free thyroxine and free triiodothyronine levels at 3.7 pg/mL and 0.8 ng/dL, respectively. A neck ultrasound (US) demonstrated left thyromegaly with a heterogeneous solid-cystic 5.5 x 3.6 x 3.8 cm left thyroid nodule, Thyroid Imaging Reporting and Data System (TIRADS) 5, and a solid hypoechoic 1.0 cm right upper lobe thyroid nodule with TIRADS 4 appearance (**Figure 2**). No suspicious cervical lymph nodes were visualized. A thrombus was noted within the left internal jugular vein (**Figure 2**). The patient was referred to hematology-oncology service and initiated on apixaban. Fine needle aspiration (FNA) of bilateral dominant nodules returned benign cytology within the left thyroid nodule and atypia of undetermined significance/Bethesda-III within the right thyroid nodule. Subsequent molecular analysis using the ThyGeNEXT platform (Interpace Diagnostics, Inc, Parsippany, New Jersey) revealed a *BRAFV600E* point mutation within the right thyroid nodule. The patient was evaluated by endocrinology and endocrine surgery services with a dedicated ultrasound lymph node mapping study which confirmed no central or lateral cervical adenopathy. A total thyroidectomy and prophylactic central compartment cervical lymph node dissection were performed. Intraoperatively, the left lobe was noted to be firm, calcific, and grossly malignant-appearing with encasement of the left recurrent laryngeal nerve. A segment of the recurrent laryngeal nerve was resected *en bloc* with the left lobe tumor. There was no adherence or gross extension into any other structures including the left common carotid artery or left internal jugular vein, which was noted to be soft and compressible.

Surgical pathology revealed pT3aN0a bilateral multi-focal follicular-variant papillary thyroid carcinoma, with the largest foci measuring 7.0 cm. Extensive microscopic angiovascular invasion and lymphatic invasion were present. Surgical margins were involved. There were no metastases present within the five central compartment lymph nodes resected. Postoperatively, the patient underwent voice therapy and endoscopic left vocal fold augmentation with Prolaryn Plus (Merz Pharmaceuticals, Frankfurt, Germany).

**Figure 3:**
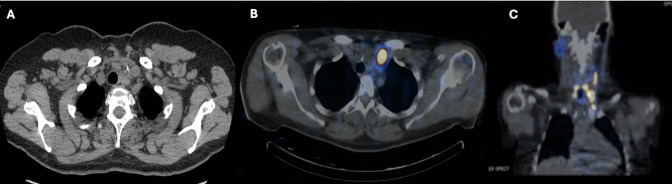
Post-thyroidectomy nuclear medicine I-123 whole-body single-photon emission computed tomography (SPECT) **(A)** axial view; **(B)**, coronal view) showing marked radiotracer uptake in the left neck extending leftward and superiorly to the level of the thyroid cartilage. Post-thyroidectomy neck CT without contrast **(C)** showed asymmetrical enlargement of the left internal jugular vein.

Due to the extent of the carcinoma, the left IJV thrombus was suspected to be malignant. Vascular surgery and radiation oncology specialists were consulted. A non-contrasted postoperative computed tomography (CT) scan of the neck and chest and an I-123 whole-body scan demonstrated asymmetrical dilation of the left IJV and proximal left subclavian vein with corresponding iodine-avidity in the I-123 whole-body scan extending superiorly toward the left posterior sternocleidomastoid at the level of the thyroid cartilage and inferiorly posterior to the left clavicular head, confirming tumor thrombus (**Figure 3**). There was no I-123 uptake reported outside of the neck. A non-contrast CT chest scan was negative for definite pulmonary metastases. The patient’s post-thyroidectomy thyroglobulin level was 4,527 ng/mL (1.3 - 31.8 ng/mL). Thyroglobulin antibodies were not detectable. A neck US did not reveal adenopathy and redemonstrated the IJV thrombus. A left IJV thrombectomy with possible resection was planned.

**Figure 4:**
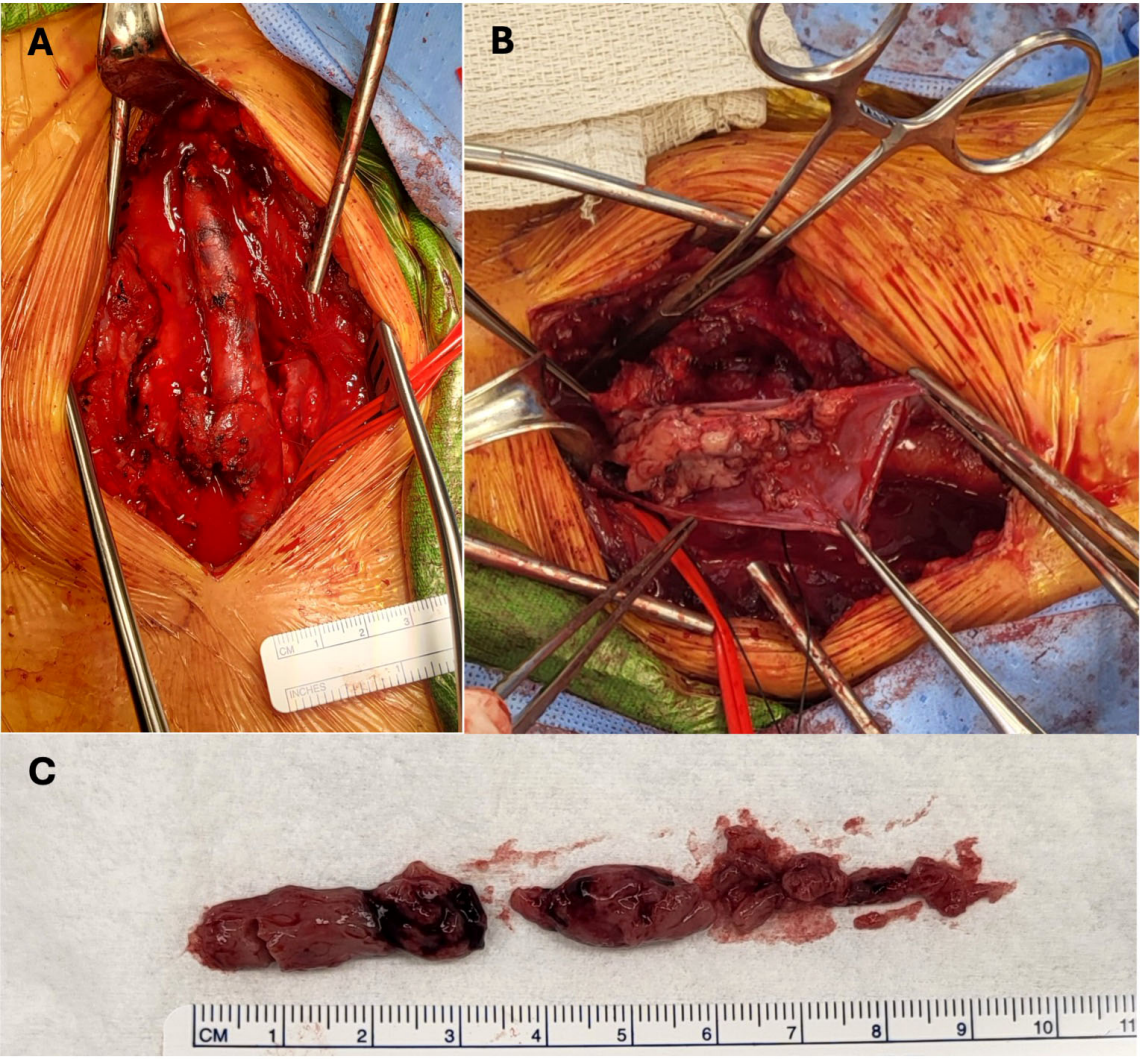
Intraoperative images demonstrating pathologically confirmed papillary thyroid cancer tumor thrombus within the left internal jugular vein post-thrombectomy **(A)** prior to thrombectomy, during resection **(B)** and *ex situ* **(C)**.

**Figure 5:**
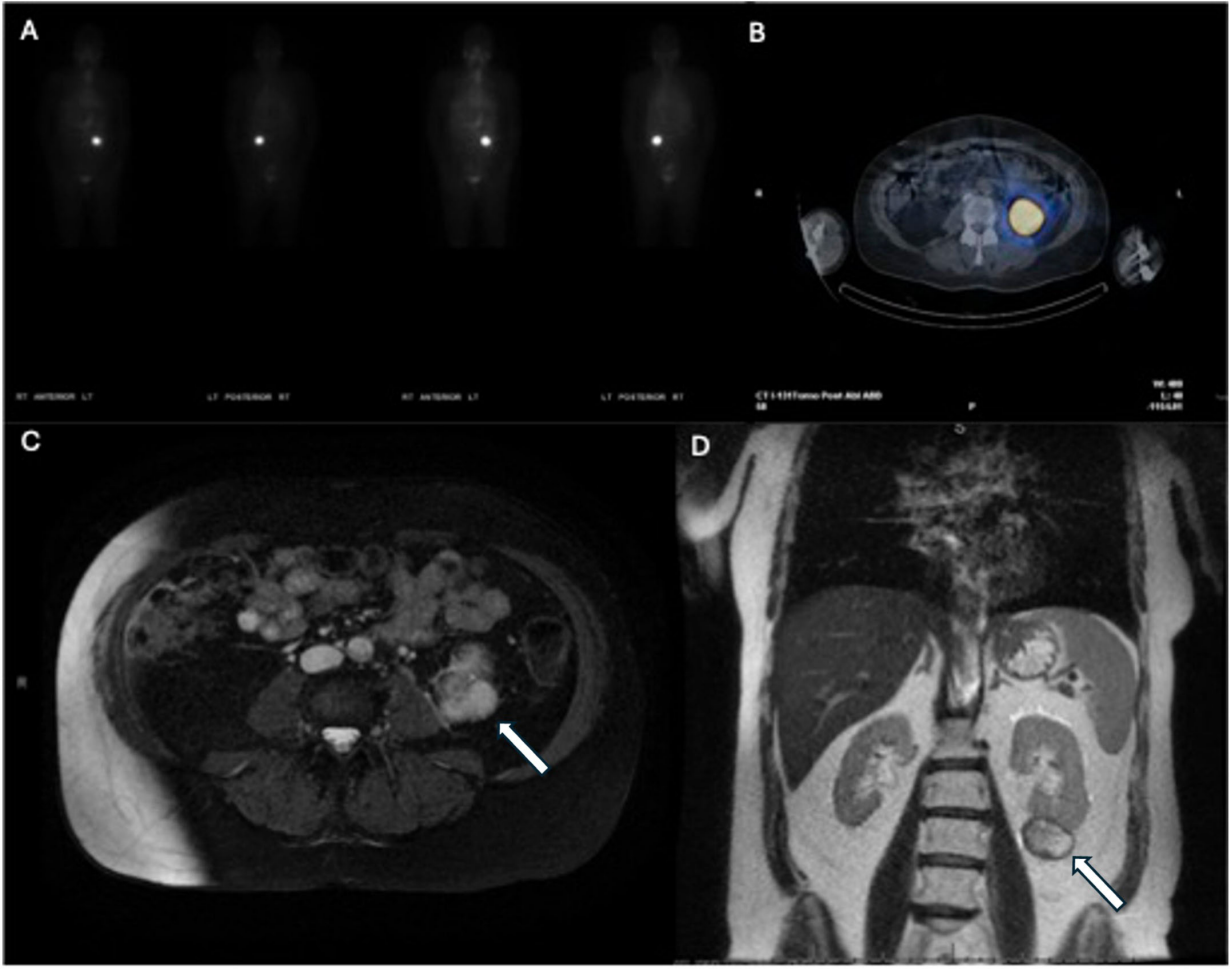
Post-radioactive iodine ablation I-131 whole-body scan **(A)** and axial view **(B)** demonstrating increased uptake in the area of the left kidney. Subsequent T2-weighted axial **(C)** and T1-weighted coronal **(D)** MRI demonstrating the left kidney mass (arrows).

Intraoperatively, the tumor thrombus was found to extend from the angle of the left mandible into the junction of the subclavian and innominate veins (**Figure 4**). An irregular lymph node was visible near the mid-left internal jugular vein. All tumor thrombus extending proximally and distally was removed and the left internal jugular vein was resected. A left lateral compartment (levels II-IV) cervical lymph node dissection was completed concurrently. Surgical pathology confirmed metastatic papillary thyroid carcinoma within intraluminal left IJV thrombus and in 1 of 37 left lateral lymph nodes. No extranodal extension was present. Postoperative thyroglobulin levels remained elevated at greater than 450 ng/mL. The patient subsequently underwent 190.5 mCi RAI I-131 treatment. An I-131 post-ablation uptake scan revealed residual intense uptake within the left neck and foci in the left kidney (**Figure 5**). The patient’s post-ablation thyroglobulin level was 13.2 ng/mL (1.6 - 50.0 ng/mL). A post-thrombectomy neck US did not show abnormal thyroidectomy bed tissue nor locoregional adenopathy. In the multidisciplinary oncology panel discussion, significant left lateral neck uptake with elevated thyroglobulin levels was concerning for persistent microscopic disease. The patient was subsequently treated with 66 Gy in 33 2 Gy daily fractions using 6 MV photons using intensity-modulated radiation therapy treatment planning and delivery techniques with a Varian TrueBeam System (Varian Medical Systems, Inc., Palo Alto, CA). She tolerated radiation without adverse events or toxicity. Concurrently, the left renal uptake was further evaluated with a dedicated US and MRI which confirmed a solid mass measuring 3.1 x 3.9 x 3.2 cm within the lower pole of the left kidney (**Figure 5**). A left robotic partial nephrectomy was performed. Metastatic papillary thyroid carcinoma with elements of angiomyolipoma was confirmed on the final pathology. Following a partial nephrectomy, serum thyroglobulin levels declined to 4.7 ng/mL (1.6 - 50.0 ng/mL).

Currently, over 2 years after the initial diagnosis, the patient is alive and without signs of structurally persistent disease. Neck ultrasound, whole-body I-123, and PET-CT studies within the past 6 months were negative for residual or metastatic disease. Her most recent unstimulated thyroglobulin level was 1.7 ng/mL (1.6 - 50.0 ng/mL).

## Systematic review results

A total of 21 unique patients were included in the analysis presented in **Table 1**, with summary descriptive statistics in **Table 2**. The majority of the patients (14, 70%) were women and the mean age was 57. Our patient, along with one other case, were the only two who presented with hypothyroidism or subclinical hypothyroidism of only 7 other cases which reported preoperative thyroid function status.

**Table 2:** Demographic features, clinical characteristics, and outcomes reported in the cohort, total n = 21.

	#/Mean	Standard Deviation	%	Range
Age (years)	57.8	15.95		24-79
Female	14		70.0	
Male	6		30.0	
Type of tumor thrombus				
Venous	20		95.2	
Arterial	1		4.8	
Caval or atrial thrombus	4		19.0	
Primary tumor size (cm)	4.22	2.64		0.9 - 8.0
Nodal metastases	12		85.7	
Distant metastases	5		25.0	
Sites of metastases	Kidney, brain, lung, bone			
Timing of thrombus diagnosis				
Preoperative	9		42.9	
Intraoperative	1		4.8	
Postoperative	11		52.4	
Thrombus diagnostic modality				
Ultrasound	8		38.1	
CT	13		61.9	
MRI	2		9.5	
18-FDG PET	2		9.5	
131-Iodine scintigraphy	3		14.3	
Transesophageal echocardiogram	2		9.5	
Treatment				
Tumor thrombectomy	10		52.6	
Vascular resection	12		66.7	
Central vessel or atrial reconstruction	8		42.1	
External beam radiation	4		21.1	
Radioactive iodine ablation	7		36.8	
Thyroglobulin levels				
Preoperative (ng/mL)	10,662	13,997.75		normal^1^ - 38,000
Postoperative (ng/mL)	204	144.7		normal^1^ - 450
Known outcome				
Recurrent disease	5		35.7	
Mortality	6		35.3	

CT, computed tomography; US, ultrasound; MRI, magnetic resonance imaging; FDG PET, fludeoxyglucose-18 positron emission tomography; ^1^”normal” reported in study text, no quantitative measurement noted.

All the reviewed cases involved primary tumors with a histopathological diagnosis of PTC, with the exception of one case with poorly differentiated thyroid carcinoma, however, the tumor thrombosis was papillary thyroid carcinoma histologically (17). Primary thyroid tumor size ranged from 0.9 cm to 8.0 cm with a mean of 4.22 cm ± 2.64 cm.

All cases involved venous thrombi, with the exception of one involving the aorta with a comorbid ascending aortic aneurysm; notably, this was the same case with the smallest primary tumor size (22). The IJV was the most commonly involved vein (71.4%), followed by the SVC (42.9%), brachiocephalic vein (BCV) (19.0%), and right atrium (4.76%%). Tumor thrombi were also found extending to the pulmonary, azygous, and intercostal veins in one case (20), the middle thyroid vein in another (12), and the axillary vein was involved in another case (27).

Molecular oncogenic information was very limited in the series as it was reported in only three cases including our own. A *BRAFV6500E* point mutation was found on initial cytopathological fine needle aspirate analysis our case. Two cases in the series did not have malignancy with associated *BRAFV600E* mutations (23) with one case additionally reporting no genetic variation in the *BRAFK601, TERT, KRAS, NRAS, EIFIAX*, or *RET* genes nor were the following fusion mutations detected: *PAX8/PPARγ, RET/PTC1*, or *RET/PTC3* (12).

Nodal metastasis was present in 12 (85.7%) cases and extrathyroidal disease (thyroid capsular invasion or involvement of other structures) was present in 7 cases (70.0%), with the recurrent laryngeal nerve and anterior tracheal wall being the most common places of involvement. Direct invasion of the sternocleidomastoid muscle occurred in one case (21). Distant metastases were present in five cases (27) with only the lungs affected in two patients (13, 24); the brain, lungs, and liver in another (23); and the ipsilateral kidney in our case.

In nine cases, tumor thrombus was diagnosed pre-thyroidectomy, with six patients presenting with SVC/thoracic outlet syndrome. No patients had a palpable thrombus on exam. Tumor thrombus was identified intraoperatively in one case (20). Tumor thrombus was diagnosed postoperatively in 11 cases, with the time from thyroidectomy ranging from 1 month to 30 years with a mean of 72.6 months and median 7 months.

In 13 cases (61.9%), tumor thrombus was diagnosed on neck CT. Sonography was the diagnostic modality in 8 cases (38.1%). Other imaging modalities included fludeoxyglucose (FDG)-18 PET (9.5%), 131-Iodine whole-body scintigraphy (14.3%), and transesophageal echocardiogram (9.5%). Several cases employed multiple diagnostic imaging modalities.

Pre-thrombectomy serum thyroglobulin levels were tested in 10 cases with a mean level of 10,662 ng/mL and were normal in three cases. Among patients with preoperative tumor thrombosis detection, the mean pre-thrombectomy thyroglobulin level was 19,069 ng/mL and ranged from within normal limits to 38,000 ng/mL.

The initial tumor thrombus management for all patients was open surgery. One patient in the series declined surgical intervention. Ten patients were treated with open thrombectomy of the affected vessel. Complete vascular resection was completed in 12 cases, eight of which involved the IJV and six also involved the SVC. Eight cases required vascular reconstructions. One patient required surgery with a partial resection of the right atrium with cardiopulmonary bypass (13).

Post-thrombectomy management was not consistently described in the cohort. Postoperatively, three patients received adjuvant RAI alone and three also went on to receive external beam radiation therapy. One patient received chemotherapy in addition to RAI and external beam radiation therapy following surgery (23).

Postoperative serum thyroglobulin levels were reported in six cases. Thyroglobulin levels normalized in one patient (18) and among the remaining five patients, the mean level was 204 ± 144.7 ng/mL. In the patients with persistently elevated thyroglobulin levels, two had persistent local disease (15, 23), three had nodal disease (15, 23), and two had metastatic disease (23).

In 10 cases, survival data was available and the mean and median postoperative time in this cohort was 14.1 ± 10.1 months. Mortality was reported in six cases during this period. Three patients died from disease progression; all had distant metastases. Among the 11 patients that underwent complex vascular resection or reconstruction (procedures involving more than simple thrombectomy), three died from respiratory failure-two 1 month postoperatively and the other 18 months after initial resection.

## Discussion

Tumor thrombi formation in PTC is a rare and poorly understood event. There is little information regarding the underlying risk factors and mechanisms to inform clinical risk assessment and pretest probability to aid timely diagnosis. Untreated thrombosis can lead to propagation requiring invasive surgical management, SVC syndrome, pulmonary embolism, and, in extremely rare arterial thrombosis, potentially fatal embolic events.

When compared to its follicular and anaplastic carcinoma countertypes (which are associated with a higher propensity for nodal involvement, hematological spread, and metastases), PubMed searches with the same search criterion used for our systematic review, unexpectedly, yielded fewer relevant articles, n = 16 and 5 respectively. In our series, we did not observe high rates of lateral or metastatic disease at the time of tumor thrombus diagnosis, suggesting distinct processes may be occurring.

There was not a single PTC thrombosis case in our series which described co-existing direct invasion of the wall of the vein or artery, again supporting a differing mechanism than that seen in other carcinomas. The disease process most likely involves lymphatic channels, with a lymphovascular spread from circulating tumor cells as tumor thrombi have been observed in vessels close to the primary tumor (8–13, 15–26). Extensive microscopic lymphovacscular invasion was found in our patient.

The combination of an unclear pathogenesis, uncertain risk factors, and a variable clinical presentation makes detecting PTC tumor thrombosis incredibly difficult. The demographic profile of the cases in the cohort did not demonstrate a distinct trend. The predominance of women with thrombosis is similar to the sex-disparate incidence rate for papillary thyroid carcinoma. The mean age was slightly higher than that seen in papillary thyroid carcinoma, but spanned seven decades. We examined hypothyroid state in the cohort as there may be increased risk for thrombosis via a TSH-dependent increase in endothelial cell dysfunction that that could predispose patients to tumor thrombosis (19, 29–31). While subclinical hypothyroidism was observed in our patient, we did not see high rates of hypothyroidism in the cohort. However, this data point was underreported.

In our series, patient clinical presentation ranged from SVC syndrome with unilateral arm and breast pain and engorgement to being completely asymptomatic, with the majority of patients being asymptomatic. Among the cohort, tumor thrombus was found to be present at the time of primary disease diagnosis or could occur decades later (27). The clinical stage or extent of disease did not appear to correlate with the development of tumor thrombosis as we observed it occurring in patients with tumors of variable sizes, including microcarcinoma, in the cohort. Notably, central compartment nodal metastases were present in nearly all patients while lateral neck disease was less common. The distant metastasis rate was similar to the general incidence.

Tumor thrombus can also be mistaken for venous thromboembolism (VTE) sonographically, which has increased frequency in patients with PTC (35) and hypothyroidism (28, 29). Liu and colleagues report two cases of patients with PTC that developed venous thrombi, however, both patients had resolution of the thrombi with oral anticoagulant treatment alone (36). Evaluation of patients with non-resolving thrombi is warranted.

Tumor thrombus was diagnosed in the majority of patients on contrasted neck CT, which is not frequently employed in the typical diagnostic workup for differential thyroid carcinoma (37). CT neck/chest evaluations may need to be considered in any patient with signs of vascular thrombosis and preoperative indeterminate-cytology, as in our patient, or malignant-cytology nodule. Preoperative serum thyroglobulin measurement is a possible tool that could be informative in some cases but its utility is unclear in our review. Preoperative serum thyroglobulin levels were normal in three cases, with approximately a third of the patients tested, but when elevated, it was distinctly marked and appeared highest in patients with more extensive, central thrombosis (i.e., brachiocephalic vein involvement). Of note, thyroglobulin elevation also occurred in those with thrombotic disease without distant metastases. Preoperative thyroglobulin levels could aid recognition in some cases, where VTE versus tumor thrombosis is unclear (i.e., low-risk cytology) or in patients with low pretest probability for malignant thrombus (i.e., a patient with strong risk factors favoring VTE, for example, Factor V Leiden) and may supplement imaging diagnostics.

Ongoing reporting is needed to understand the optimal treatment for PTC tumor thrombus. Curative measures should be utilized, as tumor thrombi—particularly in the rarest arterial cases—pose an extreme risk due to their central location. In our series, surgical treatment of tumor thrombus was the primary intervention and about half were treated with additional therapies. Short-term mortality was not observed in most patients and was absent with localized, single-vessel thrombus. Surgical complications were not always reported but did occur in some cases. One case described a postoperative bronchial infection with *Pseudomonas aeruginosa* (13), and another mentioned respiratory and cardiac failure, attributed to the patient’s chronic obstructive pulmonary disease, tricuspid regurgitation, and congestive heart failure comorbidities (20); these complications led to patient deaths in the short postoperative period. Data gaps in the systematic review limit our evaluation of treatment efficacy but do demonstrate the range of procedures and adjuvant therapies currently employed in the management of the disease.

## Limitations

Limitations of the systematic review include gaps in data points collected, a lack of detailed reporting (i.e., thyroid function status, histological details, and adjuvant treatment), and a short longitudinal follow-up period. Inconsistent reporting and a small cohort impact the reliability and generalizability of the pooled results. Risk factors, diagnostics and treatment outcomes cannot be adequately assessed to guide best practices in the diagnosis and management of the disease. Despite data deficits, it is clear that there is heterogeneity in disease presentation, which highlights the need to consider this pathology when encountering VTE in patients with cytologically indeterminate and malignant nodules and in the post-treatment/long-term surveillance period. Timely multi-disciplinary involvement is necessary for treatment planning (20).

## Conclusion

PTC vascular tumor thrombus is a rare complication of PTC with unknown associated risk factors and pathogenesis. The clinical presentation and timing of the thrombosis event vary widely. Thrombosis can be single-vessel or extensive, involving major extracervical vascular reconstructions for complete tumor clearance, and was often treated with multimodal adjuvant therapies in our case cohort. Definitive treatment is required to prevent complications from disease progression including superior vena cava syndrome or fatal embolization. Given the multidisciplinary and complex management presented in some cases, early recognition is prudent. Further reporting of these rare cases may aid in our understanding of risk factors and propensity for pathogenesis and distant metastasis and inform strategies for early diagnosis and optimal treatment.
